# The association between metabolic syndrome and major adverse cardiac and cerebrovascular events in patients with acute coronary syndrome undergoing percutaneous coronary intervention

**DOI:** 10.1038/s41598-024-51157-w

**Published:** 2024-01-06

**Authors:** Kaveh Hosseini, Amirmohammad Khalaji, Amir Hossein Behnoush, Hamidreza Soleimani, Saghar Mehrban, Zahra Amirsardari, Kimia Najafi, Mehrshad Fathian Sabet, Negin Sadat Hosseini Mohammadi, Shayan Shojaei, Farzad Masoudkabir, Hassan Aghajani, Mehdi Mehrani, Hadie Razjouyan, Adrian V. Hernandez

**Affiliations:** 1 Cardiac Primary Prevention Research Center, Cardiovascular Diseases Research Institute, Tehran University of Medical Sciences, Tehran, Iran; 2 Tehran Heart Center, Cardiovascular Diseases Research Institute, Tehran University of Medical Sciences, Tehran, Iran; 3 School of Medicine, Tehran University of Medical Sciences, Tehran, Iran; 4 Non-Communicable Diseases Research Center, Endocrinology and Metabolism Population Sciences Institute, Tehran University of Medical Sciences, Tehran, Iran; 5 Cardiogenetic Research Center, Rajaie Cardiovascular Medical and Research Center, Iran University of Medical Sciences, Tehran, Iran; 6 Hakim Children Hospital, Tehran University of Medical Sciences, Tehran, Iran; 7 School of Medicine, Babol University of Medical Sciences, Babol, Iran; 8 Department of Medicine, Penn State College of Medicine, Hershey, PA USA; 9 Health Outcomes, Policy, and Evidence Synthesis (HOPES) Group, University of Connecticut School of Pharmacy, Storrs, CT USA; 10 Unidad de Revisiones Sistemáticas y Meta-Análisis (URSIGET), Vicerrectorado de Investigación, Universidad San Ignacio de Loyola, Lima, Peru

**Keywords:** Cardiology, Interventional cardiology, Metabolic syndrome, Obesity

## Abstract

Metabolic syndrome (MetS) poses an additional risk for the development of coronary artery disease and major adverse cardiac and cerebrovascular events (MACCE). In this study, we investigated the association between MetS and its components and MACCE after percutaneous coronary intervention (PCI) in patients with acute coronary syndrome (ACS). The presence of MetS was calculated at baseline using the NCEP-ATP III criteria. The primary outcome was MACCE and its components were secondary outcomes. Unadjusted and adjusted Cox Regression models were used to calculate hazard ratios (HRs) and 95% confidence intervals (CI) of the association between MetS or its components and MACCE and its components. A total of 13,459 ACS patients who underwent PCI (MetS: 7939 and non-MetS: 5520) with a mean age of 62.7 ± 11.0 years (male: 72.5%) were included and median follow-up time was 378 days. Patients with MetS had significantly higher MACCE risk (adjusted HR [aHR] 1.22, 95% CI 1.08–1.39). The only component of MACCE that exhibited a significantly higher incidence in MetS patients was myocardial infarction (aHR 1.43, 95% CI 1.15–1.76). MetS components that were significantly associated with a higher incidence of MACCE were hypertension and impaired fasting glucose. Having three MetS components did not increase MACCE (aHR 1.12, 95% CI 0.96–1.30) while having four (aHR 1.32, 95% CI 1.13–1.55) or five (aHR 1.42, 95% CI 1.15–1.75) MetS components was associated with a higher incidence of MACCE. MetS was associated with a higher risk of MACCE in ACS patients undergoing PCI. Among MACCE components, myocardial infarction was significantly higher in patients with MetS. Impaired fasting glucose and hypertension were associated with a higher risk of MACCE. Identifying these patterns can guide clinicians in choosing appropriate preventive measures.

## Introduction

Metabolic syndrome (MetS), defined as a constellation of traditional risk factors responsible for the development of coronary artery disease (CAD)^1^, has several clinical criteria^2–6^; including central obesity, high blood pressure, high fasting plasma glucose (FPG) levels, high serum triglycerides (TG), and reduced levels of high-density lipoprotein cholesterol (HDL-C) are considered as most important factors endorsed in different definitions of MetS^7^. The prevalence of MetS has been suggested to be 39.2% in patients undergoing percutaneous coronary intervention (PCI)^8^. Although each of these risk factors individually increases the risk of CAD, MetS independently poses an additional risk for the development of CAD and major adverse cardiac and cerebrovascular events (MACCE)^2,9^.

While multiple studies have investigated the prognostic impact of MetS on the development of MACCE in patients with and without CAD^2,10,11^, the association between MetS and the clinical outcome of patients with CAD in the era of PCI is less clear. Some studies have indicated that MetS is an independent risk factor for MACCE incidence in patients with CAD undergoing revascularization^12–14^, while, some others have reported no such effect^15,16^ with one study even demonstrating a protective effect^17^. One recent study reported that MetS was associated with a greater risk of 1-year MACCE in patients undergoing PCI^18^, this risk was related to hypertension, dyslipidemia, and insulin-treated diabetes. However, other studies have shown that in patients with MetS who undergo PCI, being overweight and obese^19^ is associated with worse outcomes but the presence of diabetes is not^20^. The precise influence of MetS components on the clinical outcomes of patients with CAD undergoing PCI remains unclear. While previous research has focused on the overall impact of MetS on clinical outcomes, it is yet to be determined whether each MetS component independently or in combination with other components contributes to adverse outcomes.

In light of this debate, we aimed to assess the relationship between MetS and its components and outcomes in CAD patients who presented with acute coronary syndrome (ACS) and underwent PCI. Additionally, we investigated whether the outcome was more closely associated with a single component or a combination of MetS components.

## Methods

### Study design and population

In this retrospective cohort study, we enrolled 13,459 consecutive patients with ACS who underwent PCI at Tehran Heart Center from January 2015 to December 2021. Patients with ACS comprised ST-elevation myocardial infarction (STEMI) and non-ST elevation ACS (NSTE-ACS). NSTE-ACS includes unstable angina (UA) and non-ST elevation myocardial infarction (NSTEMI)^21^. The diagnosis of ACS was made according to the latest guidelines^22^. We excluded patients with (1) chronic coronary syndrome (CCS), (2) lack of data (i.e., hypertension, diabetes, FPG levels, waist circumference (WC), HDL-C levels, TG levels) for defining MetS, and (3) patients without follow-up after the PCI procedure. The informed consent waiver was obtained from the ethical board of the Tehran Heart Center due to the retrospective design of this study and the use of the patients’ data anonymously. The protocol of this study was approved by the Committees of Research and Ethics at Tehran Heart Center (IR.TUMS.THC.REC.1399.045) and it was conducted in accordance with the Declaration of Helsinki.

### Variables

Demographic data including age, sex, body mass index (BMI), left ventricular ejection fraction (LVEF), WC, MetS, traditional coronary risk factors (hypertension, diabetes mellitus, cigarette smoking, dyslipidemia, family history of CAD), comorbidities (heart failure, atrial fibrillation, valvular heart disease, peripheral vascular disease (PVD), chronic lung disease), past cardiovascular history (previous PCI, coronary artery bypass grafting (CABG) surgery, STEMI, NSTE-ACS, CCS, and stroke), lifestyle habits and medications history (aspirin, P2Y12 inhibitors, warfarin, statins, angiotensin-converting enzyme [ACE] inhibitors/angiotensin II receptor blockers [ARBs], beta-blockers, calcium channel blockers, and nitrates) were retrospectively gathered. Blood samples were collected at admission to determine hemoglobin, creatinine, FPG, total cholesterol, low-density lipoprotein cholesterol (LDL-C), HDL-C, and TG levels. PCI characteristics including pre-procedure Thrombolysis in Myocardial Infarction (TIMI) flow, number of occluded coronary vessels (single-vessel disease, two-vessel disease, and three-vessel disease), ACC/AHA category of the target lesion, PCI location of the target lesion, pre-procedure coronary stenosis degree, and proportion of complete revascularization of the target lesion were measured and reported.

### Definition of exposure: metabolic syndrome

Patients were categorized according to the presence or absence of MetS at baseline using the following modified criteria of NCEP-ATPIII^23^. MetS was defined as the presence of three or more of the following: (1) obesity (WC ≥ 102 cm for males, ≥ 88 cm for females); (2) impaired fasting glucose (diagnosed diabetes mellitus, FPG ≥ 100 mg⁄dl or receiving antidiabetic treatment); (3) hypertension (systolic blood pressure ≥ 130, diastolic blood pressure ≥ 85 mm Hg, diagnosed hypertension or receiving antihypertensives); (4) fasting TG ≥ 150 mg/dl; and (5) HDL-C < 40 mg/dl in men or < 50 mg/dl in women. Patients who had at least three of these five criteria were regarded as having MetS. Patients with MetS were subsequently divided into three groups according to the presence of three, four, or five criteria. Based on the MetS components combinations, patients with MetS were further categorized into sixteen subgroups as follows: Patients with three or four criteria of MetS based on NCEP-ATP III were categorized into ten and five subgroups respectively. Patients who fulfilled five criteria were also included in a separate subgroup.

### Primary and secondary outcomes

The primary outcome of the study was MACCE, defined as a composite of all-cause mortality, myocardial infarction, target lesion revascularization (TLR), target vessel revascularization (TVR), CABG, and stroke/cerebrovascular events, as used in previous studies at the Tehran Heart Center^24^. The secondary outcomes were individual MACCE components.

### Statistical analysis

Continuous variables were expressed as mean ± SD and were compared using the student’s t-test. Categorical data were presented as frequencies and percentages, and compared using the chi-square test or Fisher’s exact test. Survival between patients with MetS and those without MetS was compared using the Kaplan–Meier method and the log-rank test. Survival analysis curves were also constructed to compare outcomes according to gender and presentation of ACS (STEMI or NSTE-ACS). The association between MetS and MACCE and its components was evaluated with Cox proportional hazards models and described as hazard ratios (HR) and 95% confidence intervals (CI). Both unadjusted and adjusted Cox models were used; adjusted models included age, gender, LDL-C, creatinine, hemoglobin, LVEF, smoking, opium use, family history of CAD, and past medical history of STEMI, NSTE-ACS, CCS, atrial fibrillation, heart failure, valvular heart disease, CVA, chronic lung disease, PVD, previous CABG, and previous PCI. Moreover, in patients with MetS, adjusted and unadjusted Cox models were used to identify the effect of each MetS component on overall MACCE. A further analysis evaluated the association between having three to five MetS components and MACCE. Subgroup analyses of MACCE risk by ACS type (STEMI vs NSTEMI-ACS) were assessed with the p for interaction test; a p < 0.1 indicated a significant effect modification. A two-sided p-value of < 0.05 was considered statistically significant. All statistical analyses were performed using R software (version 4.1), utilizing packages *survival* and *survminer*.

### Ethics approval and consent to participate

This study was approved by the Committees of Research and Ethics at Tehran Heart Center (IR.TUMS.THC.REC.1399.045) and was conducted in accordance with the Declaration of Helsinki. The informed consent waiver was obtained from the ethical board of the Tehran Heart Center due to the retrospective design of this study and the use of the patients’ data anonymously.

## Results

### Patient selection

From a total of 13,682 patients undergoing PCI for ACS, 223 (1.6%) did not have follow-up data and were excluded. Of the remaining 13,459 cases, 7939 had MetS, and 5520 were in the non-MetS group were included in this study. Median follow-up was 378 days (range 313 to 589 days).

### Baseline characteristics of included patients

A detailed overview of patients’ characteristics and PCI findings is presented in Table 1. The mean age of patients with MetS was slightly higher than non-MetS ones (63.0 ± 10.6 vs. 62.1 ± 11.5, *P* < 0.001). Among the MetS group, 60.1% were male, whereas the corresponding percentage in the non-MetS group was 90.5% (*P* < 0.001). MetS patients exhibited a significantly higher prevalence of a history of PCI, CABG, CVA, STEMI, NSTE-ACS, and a family history of CAD. Non-MetS patients had a higher prevalence of cigarette smoking and opium consumption. The MetS group demonstrated higher levels of total cholesterol, TG, FPG, and creatinine. Moreover, patients with MetS had higher rates for the use of aspirin, P2Y12 inhibitors, statins, ACEi/ARB, beta-blockers, and nitrates (all *P* < 0.05).

**Table 1:** Baseline characteristics of patients undergoing PCI.

	MetS (N = 7939)	Non-MetS (N = 5520)	*P* value
Age (years)	63.0 ± 10.6	62.1 ± 11.5	< 0.001
Sex (male)	4770 (60.1%)	4994 (90.5%)	< 0.001
BMI (kg/m^2^)	29.7 ± 4.5	26.3 ± 3.7	< 0.001
Waist circumference (cm)	103.7 ± 10.1	95.4 ± 9.3	< 0.001
Hypertension	5818 (73.3%)	1415 (25.6%)	< 0.001
Diabetes	4321 (54.4%)	1076 (19.5%)	< 0.001
Cigarette smoking	2745 (34.6%)	2811 (50.9%)	< 0.001
Dyslipidemia	5879 (74.1%)	2337 (42.3%)	< 0.001
Heart failure	205 (2.6%)	154 (2.8%)	0.462
Atrial fibrillation	66 (0.8%)	37 (0.7%)	0.292
Valvular heart disease	141 (1.8%)	92 (1.7%)	0.632
Peripheral vascular disease	26 (0.3%)	16 (0.3%)	0.700
Chronic lung disease	188 (2.4%)	120 (2.2%)	0.400
Previous PCI	1510 (19%)	811 (14.7%)	< 0.001
Previous CABG	868 (10.9%)	439 (7.9%)	< 0.001
History of CVA	296 (3.7%)	129 (2.3%)	< 0.001
Family history of CAD	1614 (20.3%)	1029 (18.6%)	0.015
Opium consumption	961 (12.1%)	1133 (20.5%)	< 0.001
History of STEMI	477 (6%)	282 (5.1%)	0.026
History of NSTEMI	1106 (13.9%)	695 (12.6%)	0.025
History of UA	3023 (38.1%)	1758 (31.8%)	< 0.001
History of SA	174 (2.2%)	100 (1.8%)	0.125
LVEF (%)	46 ± 9	45.3 ± 9.2	< 0.001
Total cholesterol (mg/dL)	158.9 ± 43.7	156.4 ± 40.7	< 0.001
Triglyceride (mg/dL)	180.1 ± 113.3	120.2 ± 66.8	< 0.001
LDL-C (mg/dL)	98.3 ± 35.2	98.3 ± 34.6	0.953
HDL-C (mg/dL)	37.1 ± 8.9	41.6 ± 10.1	< 0.001
FPG (mg/dL)	141.2 ± 59.4	109.9 ± 42.4	< 0.001
Creatinine (mg/dL)	1.00 ± 0.54	0.98 ± 0.38	0.018
Hemoglobin (g/dL)	14.5 ± 1.9	15 ± 1.7	< 0.001
Lesion length (mm)	25.9 ± 13.1	25.9 ± 12.8	0.801
Pre-procedure coronary stenosis degree (%)	91.4 ± 8.9	92.1 ± 9.0	< 0.001
Drug history			
Aspirin	5993 (75.5%)	3631 (65.78%)	< 0.001
P2Y12 inhibitor	3592 (45.2%)	2390 (43.3%)	0.025
Warfarin	50 (0.6%)	49 (0.9%)	0.085
Statins	5351 (67.4%)	3231 (58.5%)	< 0.001
ACEi/ARB	5200 (65.5%)	2469 (44.7%)	< 0.001
Beta-blocker	4649 (58.5%)	2713 (49.1%)	< 0.001
Calcium channel blocker	992 (12.5%)	275 (5.0%)	< 0.001
Nitrates	4094 (51.6%)	2551 (46.2%)	< 0.001
ACS type			
STEMI	2651 (33.4)	2162 (39.2)	< 0.001
NSTEMI	1415 (17.8)	1046 (18.9)	
UA	3873 (48.8)	2312 (41.9)	
Pre-procedure TIMI flow			
0	1979 (24.9%)	1567 (28.4%)	< 0.001
1	309 (3.9%)	239 (4.3%)	
2	1085 (13.7%)	761 (13.8%)	
3	4566 (57.5%)	2953 (53.5%)	
Number of occluded coronary vessels			
Single vessel	2742 (34.5%)	2150 (38.9%)	< 0.001
Two vessels	2753 (34.7%)	1877 (34.0%)	
Three vessels	2431 (30.6%)	1478 (26.8%)	
ACC/AHA category of target lesion			
A	11 (0.1%)	10 (0.2%)	0.012
B1	1521 (19.2%)	936 (16.9%)	
B2	1091 (13.7%)	788 (14.3%)	
C	5314 (66.9%)	3785 (68.6%)	
PCI location of the target lesion			
Ostial	881 (11.1%)	597 (10.8%)	0.207
Proximal	2834 (35.7%)	2053 (37.2%)	
Non-proximal	4224 (53.2%)	2870 (52%)	
Complete revascularization of the target lesion	7533 (95.0%)	5209 (94.4%)	0.165

BMI: body mass index, PCI: percutaneous coronary intervention, CABG: coronary artery bypass grafting, CVA: cerebrovascular accident, STEMI: ST-elevated myocardial infarction, NSTEMI: non-ST-elevated myocardial infarction, UA: unstable angina, SA: stable angina, LVEF: left ventricular ejection fraction, LDL-C: low-density lipoprotein cholesterol, HDL-C: high-density lipoprotein cholesterol, FPG: fasting plasma glucose, MI: myocardial infarction, ACS: acute coronary syndrome, TIMI: thrombolysis in myocardial infarction, ACC/AHA: American College of Cardiology/American Heart Association.

The presentation with STEMI and NSTEMI was lower among patients with MetS (NSTEMI: 17.8% vs. 18.9%, STEMI: 33.4% vs. 39.2%), while UA was more frequently observed in these patients (48.8% vs. 41.9%). Pre-procedure coronary vessel stenosis degree was slightly lower among MetS patients (91.4% ± 8.9% vs. 92.1% ± 9.0%, *P* < 0.001). Although non-MetS patients had relatively lower pre-procedural Thrombolysis in Myocardial Infarction (TIMI) flow (*P* < 0.001), they had a higher prevalence of single-vessel occlusion compared to the MetS group (38.9% vs. 34.5%, *P* < 0.001). The proportion of complete revascularization of the target lesion was comparable between MetS and non-MetS groups (MetS: 95.0%, non-MetS: 94.4%, *P* = 0.165).

### Study outcomes in MetS vs. non-MetS patients

**Figure 1:**
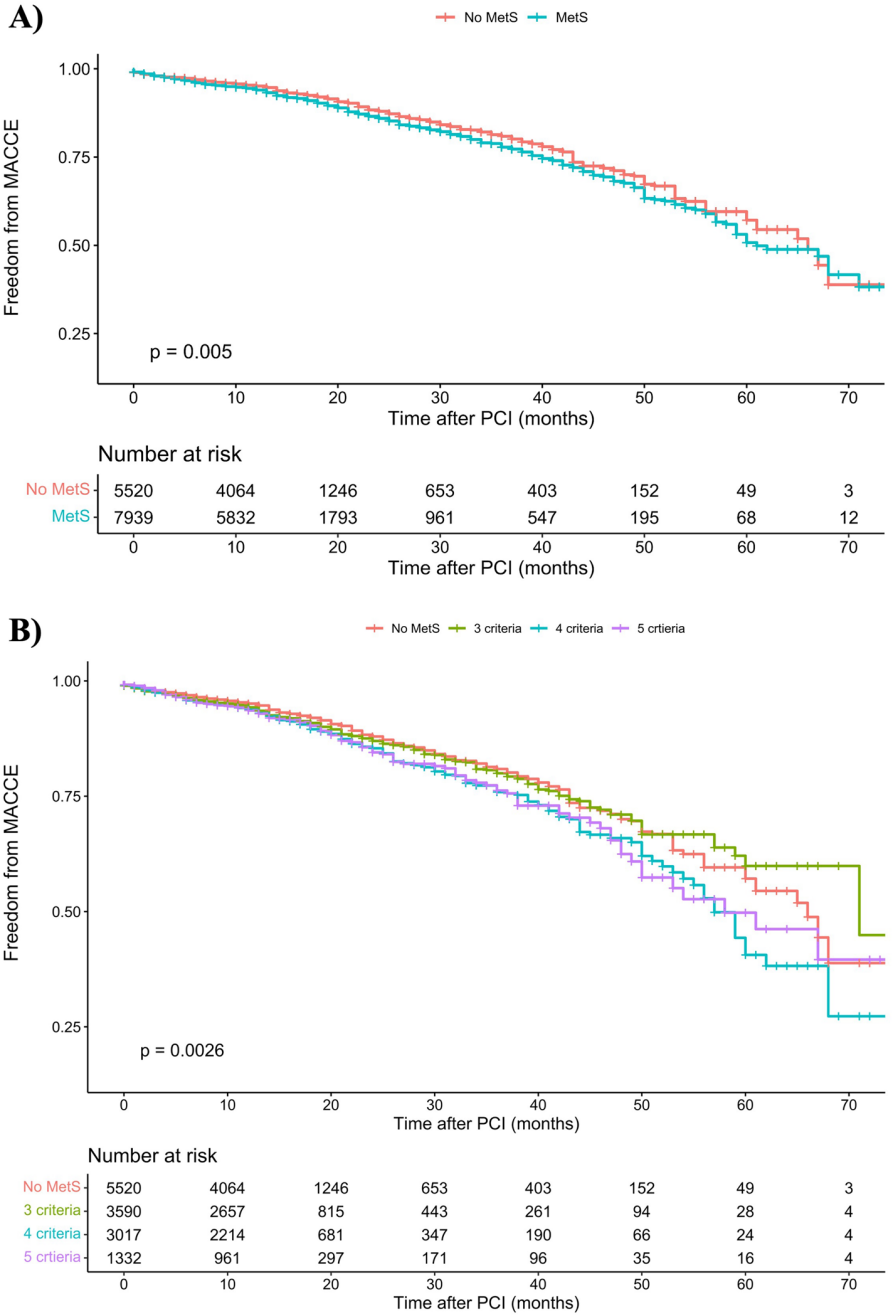
Kaplan Meier figure for comparing MACCE outcome between (**A**) MetS and non-MetS patients, and (**B**) different number of criteria for MetS; MACCE: major adverse cardiac and cerebrovascular events, PCI: percutaneous coronary intervention.

Table 2 compares outcomes occurrence in addition to HRs for MACCE and secondary outcomes between patients with MetS and non-MetS individuals. The Kaplan–Meier curve comparing MACCE between two groups is presented in Fig. 1A. The unadjusted model showed that patients with MetS had significantly higher MACCE outcomes over time compared to the non-MetS group (HR 1.175, 95% CI 1.050 to 1.316, *P* = 0.005). In line, the adjusted model found 22.4% more MACCE occurrence in MetS compared to non-MetS (adjusted HR [aHR] 1.224, 95% CI 1.077 to 1.392, *P* = 0.002).

**Table 2:** Primary and secondary outcomes comparison in patients with and without metabolic syndrome.

Outcome	Non-MetS (N = 5520)	MetS (N = 7939)	p-value
Primary outcome			
MACCE (%)			
Event (%)	478 (8.66)	810 (10.20)	0.003
Unadjusted HR [95% CI]	Ref	1.17 [1.05–1.32]	0.005
Adjusted HR [95% CI]	Ref	1.22 [1.08–1.39]	0.002
Secondary outcomes			
Coronary artery bypass grafting			
Event (%)	50 (0.90)	87 (1.10)	0.280
Unadjusted HR [95% CI]	Ref	1.21 [0.85–1.71]	0.287
Adjusted HR [95% CI]	Ref	1.29 [0.88–1.89]	0.195
Myocardial infarction			
Event (%)	165 (2.99)	301 (3.79)	0.012
Unadjusted HR [95% CI]	Ref	1.27 [1.05–1.54]	0.013
Adjusted HR [95% CI]	Ref	1.43 [1.15–1.76]	0.001
Mortality			
Event (%)	161 (2.92)	270 (3.40)	0.116
Unadjusted HR [95% CI]	Ref	1.16 [0.96–1.41]	0.126
Adjusted HR [95% CI]	Ref	1.12 [0.89–1.41]	0.340
Stroke			
Event (%)	12 (0.22)	18 (0.23)	0.910
Unadjusted HR [95% CI]	Ref	1.05 [0.51–2.18]	0.892
Adjusted HR [95% CI]	Ref	1.07 [0.44–2.58]	0.881
Target lesion revascularization			
Event (%)	51 (0.92)	78 (0.98)	0.732
Unadjusted HR [95% CI]	Ref	1.08 [0.76–1.54]	0.672
Adjusted HR [95% CI]	Ref	1.15 [0.77–1.71]	0.489
Target vessel revascularization			
Event (%)	39 (0.71)	56 (0.70)	0.994
Unadjusted HR [95% CI]	Ref	0.99 [0.66–1.49]	0.963
Adjusted HR [95% CI]	Ref	0.84 [0.52–1.34]	0.455

MACCE: major adverse cardiac and cerebrovascular events, HR: hazard ratio, CI: confidence interval, MetS: metabolic syndrome, Ref: reference.

**Figure 2:**
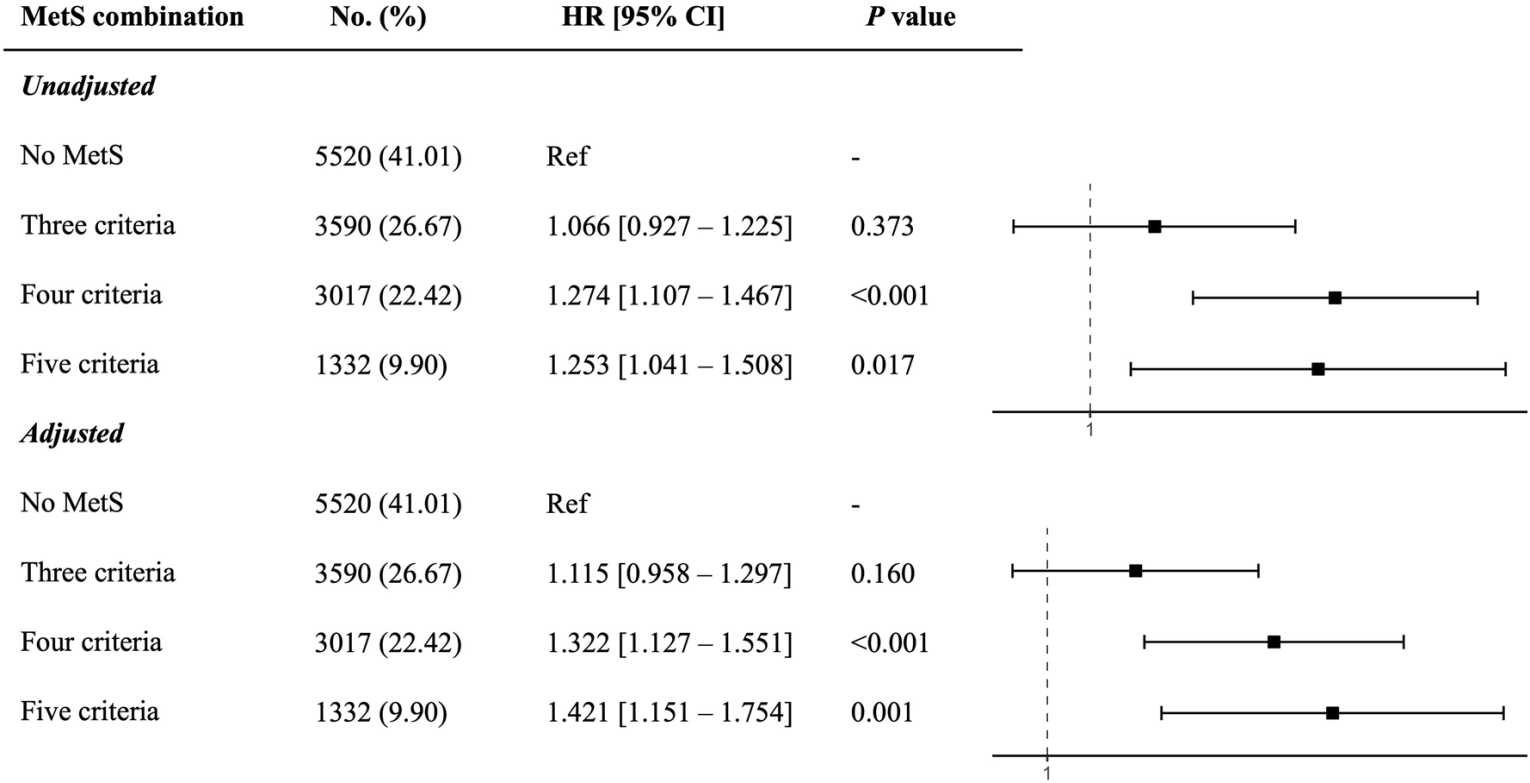
Hazard ratios for unadjusted and adjusted models for three, four, and five criteria of MetS combinations, compared to non-MetS group.

In the comparison of patients having three, four, or five criteria of MetS and patients without MetS, we found a significant difference in MACCE incidence between these groups (*P* = 0.003, Fig. 1B). We also compared the HRs of MACCE incidence in patients with three, four, or five MetS components to non-MetS patients in Fig. 2. Having three MetS components did not increase the probability of MACCE (unadjusted HR 1.066, 95% CI 0.927 to 1.255, *P* = 0.373), while having four or five MetS components was associated with a higher prevalence of MACCE, with unadjusted HRs of 1.274 [95% CI 1.107 to 1.467, *P* < 0.001) and 1.253 [95% CI 1.041 to 1.508, *P* = 0.017), respectively. These ratios further increased after adjustment (four criteria: HR 1.322, 95% CI 1.127 to 1.551, *P* < 0.001; five criteria: HR 1.421, 95% CI 1.151 to 1.754, *P* = 0.001). The HRs associated with patients having different combinations of MetS components (16 combinations) are shown in Supplementary Table 1.

The only MACCE component that exhibited a significant difference between MetS and non-MetS patients was MI, with an unadjusted HR of 1.272 [95% CI 1.052 to 1.538, *P* = 0.013] in the MetS group compared to non-MetS. This risk is higher when adjusted (aHR 1.427, 95% CI 1.155 to 1.765, *P* = 0.001). Kaplan–Meier figures for each MACCE component are illustrated in Supplementary Figs. 1–6.

### MACCE in subgroups of patients

**Figure 3:**
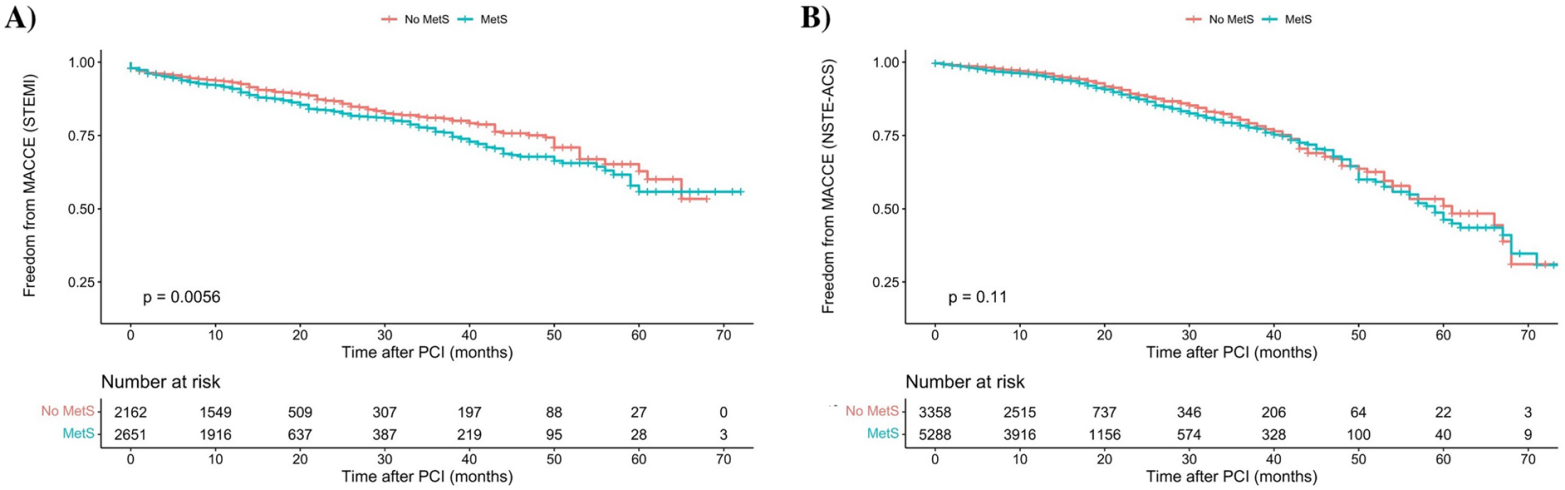
Kaplan Meier figure for comparing MACCE outcome in (**A**) patients with STEMI, and (**B**) patients with NSTE-ACS; MACCE: major adverse cardiac and cerebrovascular events, PCI: percutaneous coronary intervention.

Figure 3 shows the freedom from MACCE in subgroups of STEMI and NSTE-ACS since the p for interaction tests was significant for them (*P* < 0.001). It was shown that, in patients with STEMI, MetS patients exhibit a greater incidence of MACCE in unadjusted model (HR 1.265, 95% CI 1.07 to 1.494, *P* = 0.006, Fig. 3A) and adjusted model (aHR 1.244, 95% CI 1.026 to 1.508, *P* = 0.026), whereas this distinction is not observed among NSTE-ACS patients in unadjusted model (HR 1.135, 95% CI 0.973 to 1.324, *P* = 0.110, Fig. 3B), however in adjusted model there was significant difference in the incidence of MACCE (aHR 1.208, 95% CI 1.01 to 1.435, *P* = 0.031).

### Association between MetS components and MACCE

**Figure 4:**
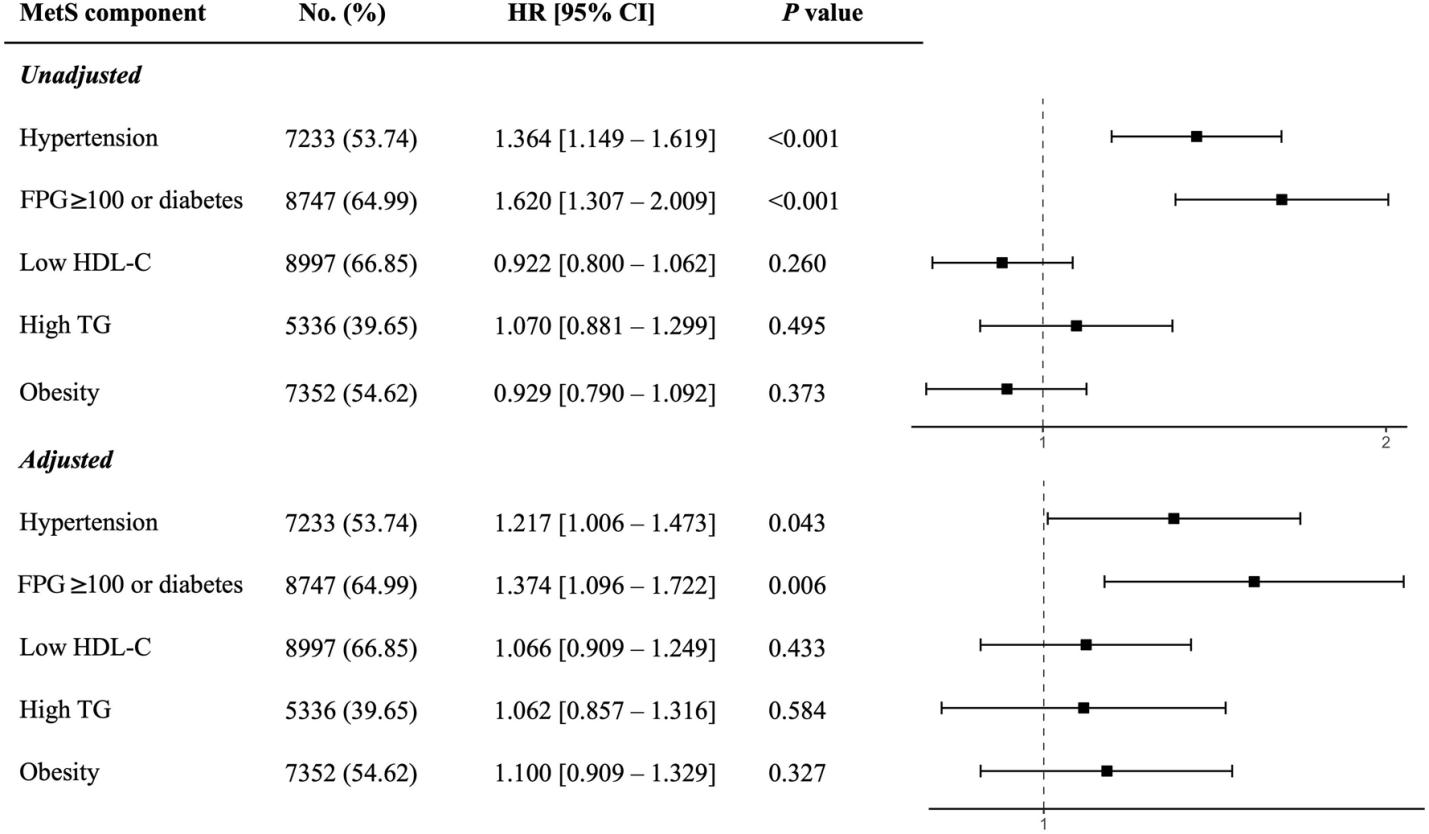
Hazard ratios for unadjusted and adjusted models for each of the MetS components; FPG: fasting plasma glucose, HDL-C: high-density lipoprotein cholesterol, TG: triglyceride.

The effects of each MetS component on MACCE are illustrated in Fig. 4. MetS components that were significantly associated with a higher incidence of MACCE were hypertension and impaired fasting glucose while no significant correlation was found between other MetS components (low HDL-C, high TG, or obesity) and MACCE. Hypertension was associated with a 36.4% increase (HR 1.364, 95% CI 1.149 to 1.619, *P* < 0.001) in the occurence of MACCE in patients who underwent PCI while the adjusted model showed a 21.7% increase (HR 1.217, 95% CI 1.006 to 1.473, *P* = 0.043). Moreover, having impaired fasting glucose was associated with higher MACCE incidence in unadjusted (HR 1.620, 95% CI 1.307 to 2.009, *P* < 0.001) and adjusted (aHR 1.374, 95% CI 1.096 to 1.722, *P* = 0.006) models.

## Discussion

In this study of the registry of ACS patients who underwent PCI at Tehran Heart Center, we found that the presence of MetS was associated with higher MACCE incidence. Although MetS is defined as having three or more of the mentioned criteria, we found worse outcomes in patients who had four or five criteria. Moreover, MetS was associated with higher MI incidence vs. absence of MetS. Finally, hypertension and impaired fasting glucose were independently associated with higher MACCE incidence while obesity, TG levels, and HDL-C levels showed no significant association with the primary outcome.

Several studies have evaluated the prognostic significance of MetS on different cardiovascular outcomes. A meta-analysis including nearly one million individuals found that MetS was associated with a two-fold and 1.5-fold increase in cardiovascular outcomes and all-cause mortality, respectively^2^. In addition to the impact of MetS on the general population, some studies investigated whether MetS can influence outcomes in patients with CAD^25–27^. In a population of premature acute MI at young age, Gao et al.^25^ found a direct association between MetS and MACE incidence. Moreover, Fanta et al.^26^ found higher in-hospital MACE and 30-day mortality in ACS patients with MetS compared with ACS patients without MetS. Similar to our study, Marso et al.^27^ investigated the prognostic value of MetS on MACE outcomes in ACS patients who underwent PCI with three years of follow-up. With a median follow-up of 3.4 years and in 37 centers in Europe and the United States, they found higher MACE incidence in patients with MetS compared to non-MetS individuals (21.3% vs. 17.4%).

Although we found a higher incidence of MACCE in this cohort who had four or five criteria of MetS, it should be noted that all the patients included in this study presented with ACS as a major cardiovascular event. Thus, ACS patients who had more risk factors, especially impaired fasting glucose and hypertension, tended to have more MACCE incidence as a secondary event after ACS and the prognostic ability of MetS in predicting first cardiovascular events should not be trivialized. Hygriv Rao et al. found significantly higher diabetes and hypertension prevalence in patients with ACS compared to healthy controls, showing their importance as predictors of ACS as the first cardiovascular event^28^.

Hypertension is a well-studied cardiovascular risk factor^29^. Patients with hypertension develop more cardiovascular diseases including CAD, atrial fibrillation, heart failure, aortic syndromes, and heart valve diseases^30^. As hypertension is a known risk factor for CAD (ACS and SA), its role in the development of atherosclerosis has been studied^31^. In addition to the higher atherosclerosis rate in hypertensive patients, shared risk factors in ACS and hypertension (e.g., diabetes, genetic susceptibility, male sex, and older age) are other reasons for increased cardiovascular outcomes in ACS patients with hypertension.

Several studies have evaluated the prognostic value of a history of hypertension in patients with ACS^32^. In a non-diabetic cohort of patients with STEMI who underwent primary PCI, no difference was found between hypertensive and non-hypertensive individuals in terms of in-hospital mortality^33^. Similar to the previous study, Cecchi et al.^34^ found comparable short- and long-term mortality between ACS patients with and without hypertension. In contrast, De Luca et al.^35^ found hypertension as a risk factor for cardiovascular outcomes in ACS patients who underwent primary PCI. They found higher mortality, reinfarction, stent thrombosis, TVR, and impaired postprocedural TIMI 0–2 in patients with hypertension compared to non-hypertensive ones. Although there is controversy regarding the exact effect of hypertension on MACCE in ACS patients, we found hypertension positively correlated with MACCE incidence in this cohort.

The effects of diabetes on cardiovascular health have been assessed comprehensively^36,37^. The prevalence of ischemic heart disease has been described to be higher in diabetic patients, resulting in higher comorbidity and mortality in these patients than in non-diabetic cases^38,39^. Moreover, increased risks of death, MI, and revascularization have been observed in diabetic patients undergoing PCI^40^. In a study by Hansen et al., the authors showed that more than half of the diabetic patients undergoing PCI had readmissions within one year, which was increased by the presence of other risk factors^41^. This finding was observed in other studies as well^42,43^. These patients which comprised 40% of our population (54.4% in the MetS group and 19.5% in the non-MetS group), are more likely to have complex multi-vessel CVDs^44^. With a cutoff of 100 mg/dL which includes prediabetic patients as well, among patients with MetS, diabetes was associated with a 37% increase in MACCE in our study. It has been previously observed that patients with prediabetes are at increased risk of all-cause mortality and cardiovascular disease in the general population and in patients with atherosclerotic disease^45^. In summary, patients with diabetes should be given special consideration in ACS conditions as it has been shown that they are also at increased risk of stroke and all-cause death even in the case of normal angiography^46^.

The five components of MetS including insulin resistance, abdominal obesity, hypertension, high TG levels, and low HDL-C levels are interrelated while seeming to be independent. For instance, hypertension might have resulted from baroreceptor impairment in obese patients while obesity is a recognized risk factor for diabetes and dyslipidemia^47^. Analysis of MetS components has been performed in several other studies^48–50^. Liu et al. found abdominal obesity and insulin resistance are two main factors of coronary collateralization (CC) in chronic total occlusion (CTO)^48^. Also, in a report from the Tehran Lipid and Glucose Study (TLGS) conducted during 20 years of follow-up, abdominal obesity, hypertension, and insulin resistance were identified as risk factors for sudden cardiac death^49^. Finally, in a single-center study conducted on 10,422 patients undergoing PCI, diabetes was identified as the only component of MetS being an independent risk factor for MACCE^50^. In our study, we found that having three MetS components did not have an effect on the incidence of MACCE, compared to the non-MetS group. However, four and five criteria were associated with increased risk. In a separate analysis of each criterion’s effect on overall MACCE in our study, the combination of insulin resistance criteria and hypertension with (1) abdominal obesity, (2) low HDL-C and high TG, (3) low HDL-C and abdominal obesity, and (4) all other three criteria were associated with increased MACCE risk in the adjusted model. The presence of hypertension and insulin resistance in all these significant groups highlights the importance of these two major risk factors. In other words, our findings suggest that metabolic syndrome can be summarized in these two criteria and special attention and follow-up should be given to hypertensive diabetic patients with ACS undergoing PCI. Lifestyle interventions should focus more on these two risk factors and it is also recommended that they be given higher weights in future models in the same settings.

In this study, we found that hypertension and diabetes were mostly responsible for higher MACCE occurrence in the MetS group compared to non-MetS. Although diabetes and hypertension are well-known risk factors for outcomes following PCI, this study highlighted the importance of these two risk factors superior to other components of MetS (high TG, low HDL-C, and high WC). The clinical relevance of this study suggests that although MetS is an independent risk factor of MACCE, diabetes and hypertension have more impact on the occurrence of MACCE, and primary care centers and clinicians should consider these two risk factors more than other components of MACCE, in the risk assessment for outcome occurrence after PCI in patients with ACS. Future studies are warranted to confirm these findings.

Although providing novel insights into the effects of MetS on PCI outcomes as well as comparing components of MetS and their impact on prognosis on a large number of patients, the current study has several limitations. First, the single-center nature of this study might threaten the generalizability of our findings which need to be confirmed in larger studies. Second, we categorized patients as MetS and non-MetS based on ATP-III criteria while there are some other definitions of MetS that might lead to different results. Third, we assessed the first MACCE only in our study and hence, the recurrence of MACCE was not considered in our analysis. It is worth mentioning that less than 1% of our population experienced second MACCE which makes it less likely to affect our overall results. Finally, inherent limitations of observational studies including the presence of confounders and selection bias limited the generalizability of our results.

## Conclusions

MetS was an independent risk factor for the incidence of overall MACCE in patients with ACS undergoing PCI. Having four or five MetS components showed higher MACCE risk than having three MetS components; MetS also was associated with an increased risk of MI. We identified that impaired fasting glucose and hypertension criterion were two MetS components associated with the incidence of MACCE. This can have clinical and policy implications in order to give more attention to these risk factors than the other three, despite having relationships with each other.

### Supplementary Information

Supplementary Information.

## Data Availability

The data used in this study will be made available upon reasonable request from the corresponding author.
