# Supplementary material for: The association between metabolic syndrome and major adverse cardiac and cerebrovascular events in patients with acute coronary syndrome undergoing percutaneous coronary intervention
Source: Sci Rep. 2024 Jan 6;14:697. doi: 10.1038/s41598-024-51157-w (PMC10771421; doi:10.1038/s41598-024-51157-w)
Supplement: Supplementary file 1 — Supplementary Information. [file 41598_2024_51157_MOESM1_ESM.docx]

***Supplementary Table 1.*** Primary and secondary outcomes comparison in patients with and without metabolic syndrome

| **MetS combination** | **Number of cases**  **(N = 13459)** | **Unadjusted HR [95% CI]** | **Adjusted HR [95% CI]** |
| --- | --- | --- | --- |
| No MetS | 5520 (41.01) | Ref | Ref |
| ①, ②, ③ | 607 (4.51) | 1.523 [1.197 – 1.938]^***^ | 1.164 [0.897 – 1.509] |
| ①, ②, ④ | 181 (1.34) | 1.186 [0.731 – 1.924] | 1.173 [0.699 – 1.969] |
| ①, ②, ⑤ | 529 (3.93) | 1.381 [1.062 – 1.796]^*^ | 1.412 [1.050 – 1.898]^*^ |
| ①, ③, ④ | 449 (3.34) | 0.868 [0.616 – 1.224] | 1.038 [0.711 – 1.516] |
| ①, ③, ⑤ | 591 (4.39) | 1.050 [0.800 – 1.379] | 1.159 [0.861 – 1.560] |
| ①, ④, ⑤ | 200 (1.49) | 1.187 [0.721 – 1.953] | 1.422 [0.832 – 2.432] |
| ②, ③, ④ | 187 (1.39) | 1.162 [0.751 – 1.800] | 1.302 [0.828 – 2.048] |
| ②, ③, ⑤ | 480 (3.57) | 0.725 [0.504 – 1.042] | 0.785 [0.536 – 1.151] |
| ②, ④, ⑤ | 77 (0.57) | 0.539 [0.173 – 1.679] | 0.856 [0.274 – 2.673] |
| ③, ④, ⑤ | 289 (2.15) | 0.492 [0.289 – 0.837]^**^ | 0.706 [0.405 – 1.232] |
| ①, ②, ③, ④ | 405 (3.01) | 1.490 [1.124 – 1.974]^**^ | 1.411 [1.038 – 1.918]^*^ |
| ①, ②, ③, ⑤ | 1340 (9.96) | 1.441 [1.205 – 1.723]^***^ | 1.337 [1.084 – 1.650]^**^ |
| ①, ②, ④, ⑤ | 347 (2.58) | 1.080 [0.738 – 1.582] | 1.143 [0.763 – 1.710] |
| ①, ③, ④, ⑤ | 592 (4.4) | 1.041 [0.788 – 1.377] | 1.277 [0.948 – 1.719] |
| ②, ③, ④, ⑤ | 333 (2.47) | 0.970 [0.667 – 1.411] | 1.339 [0.908 – 1.977] |
| ①, ②, ③, ④, ⑤ | 1332 (9.9) | 1.253 [1.041 – 1.507]^*^ | 1.413 [1.143 – 1.747]^**^ |

① Fasting blood sugar ≥ 100 mg/dl or diabetes, ②: hypertension, ③: low high-density lipoprotein cholesterol, ④: high triglycerides, ⑤: obesity

Abbreviations: MetS: metabolic syndrome, HR: hazard ratio, CI: confidence interval

*: P <0.05, **: P <0.01, ***: P <0.001


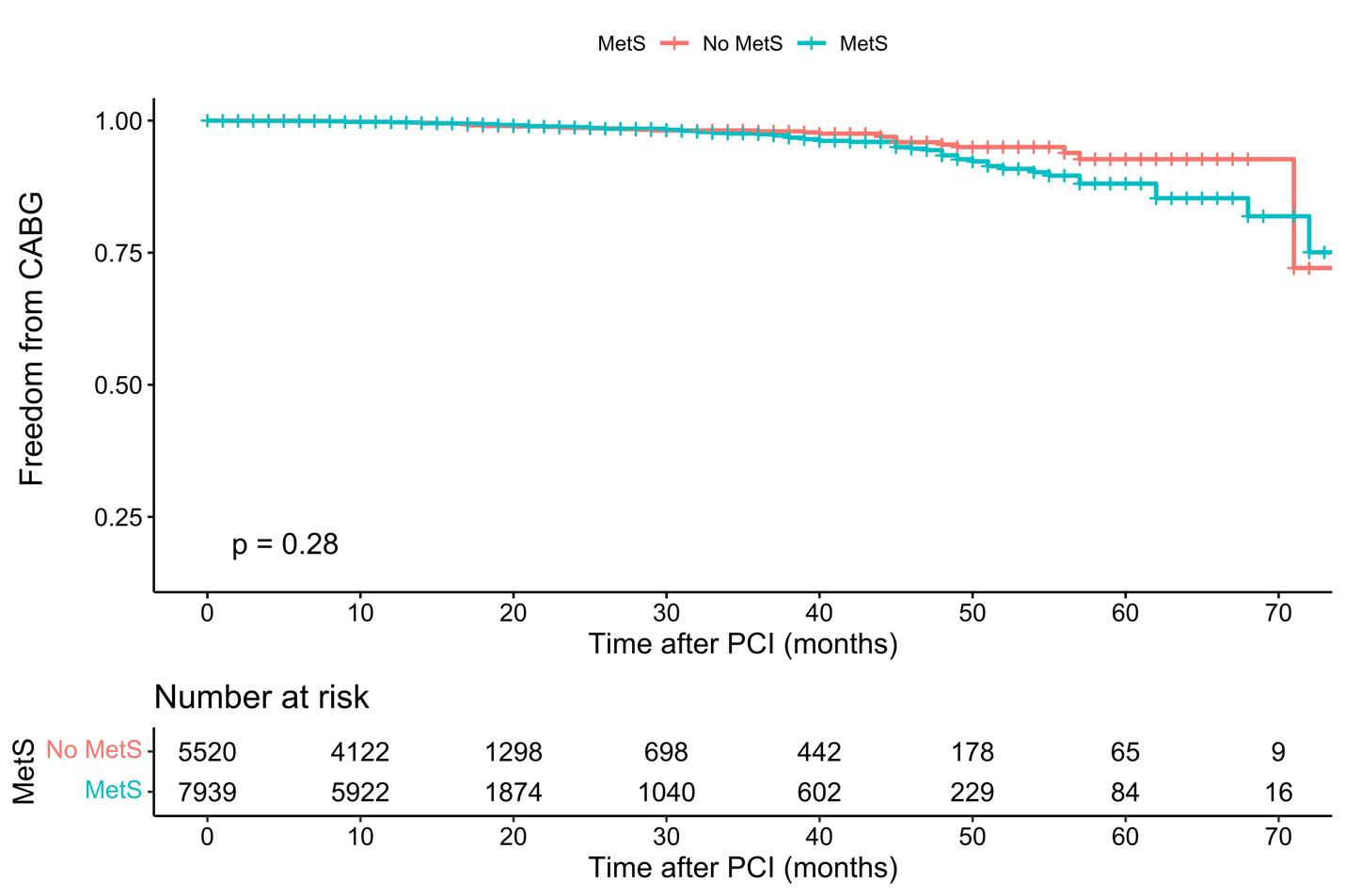


**Supplementary Figure 1.** Kaplan Meier figure for comparing coronary artery bypass grafting surgery outcomes between MetS and non-MetS patients


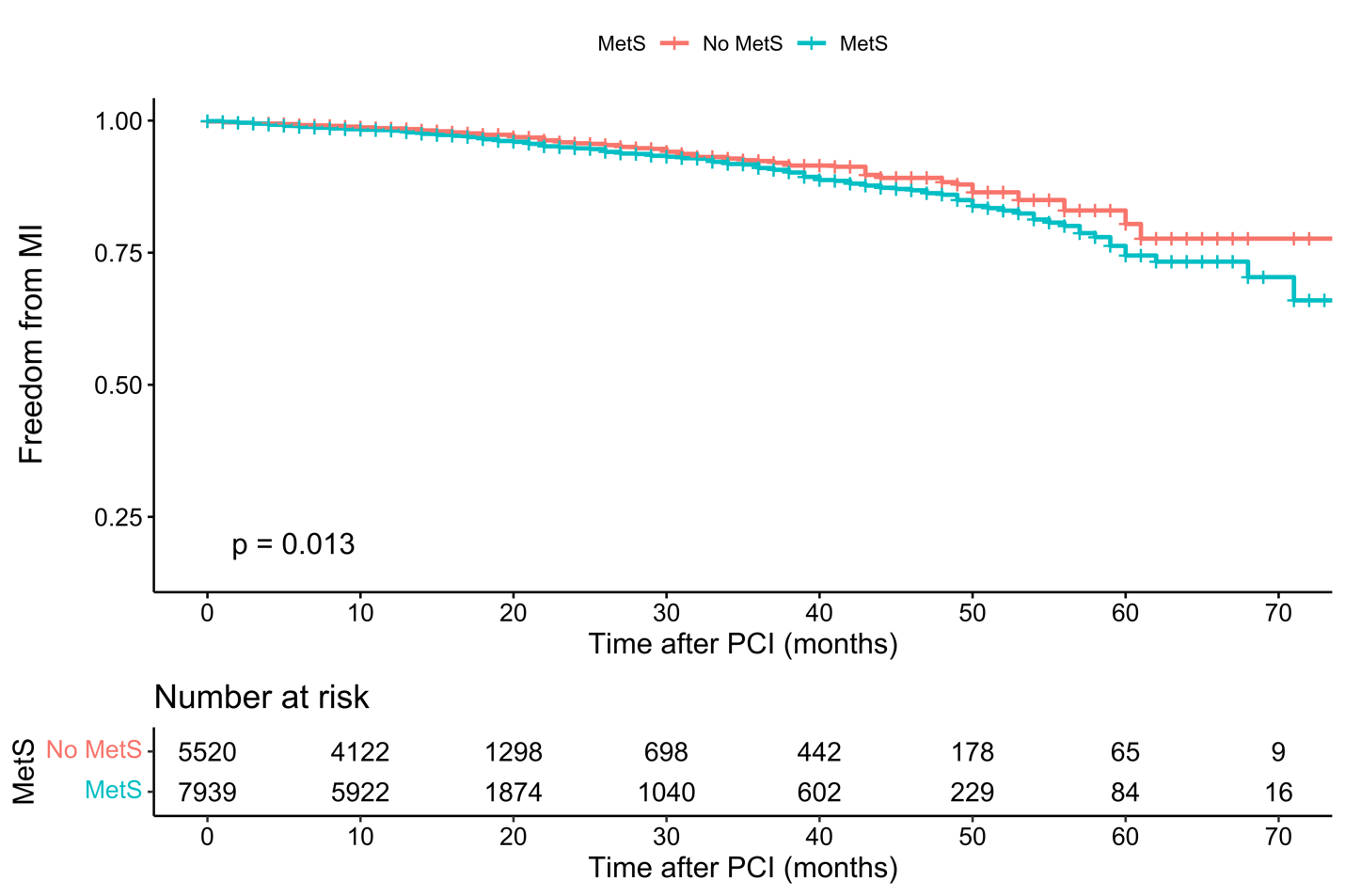


**Supplementary Figure 2.** Kaplan Meier for comparing myocardial infarction outcomes between MetS and non-MetS patients


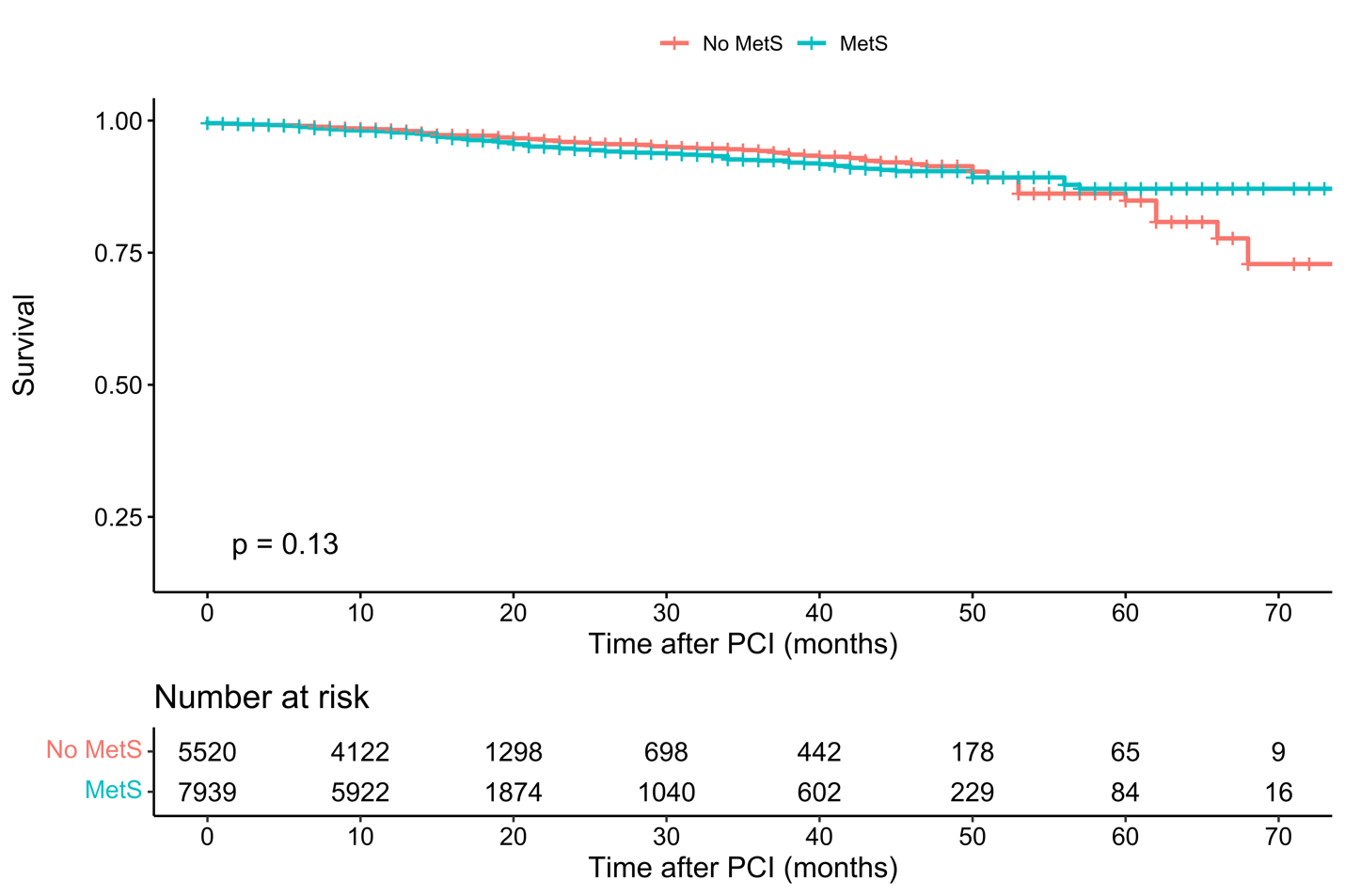


**Supplementary Figure 3.** Kaplan Meier for comparing survival between MetS and non-MetS patients


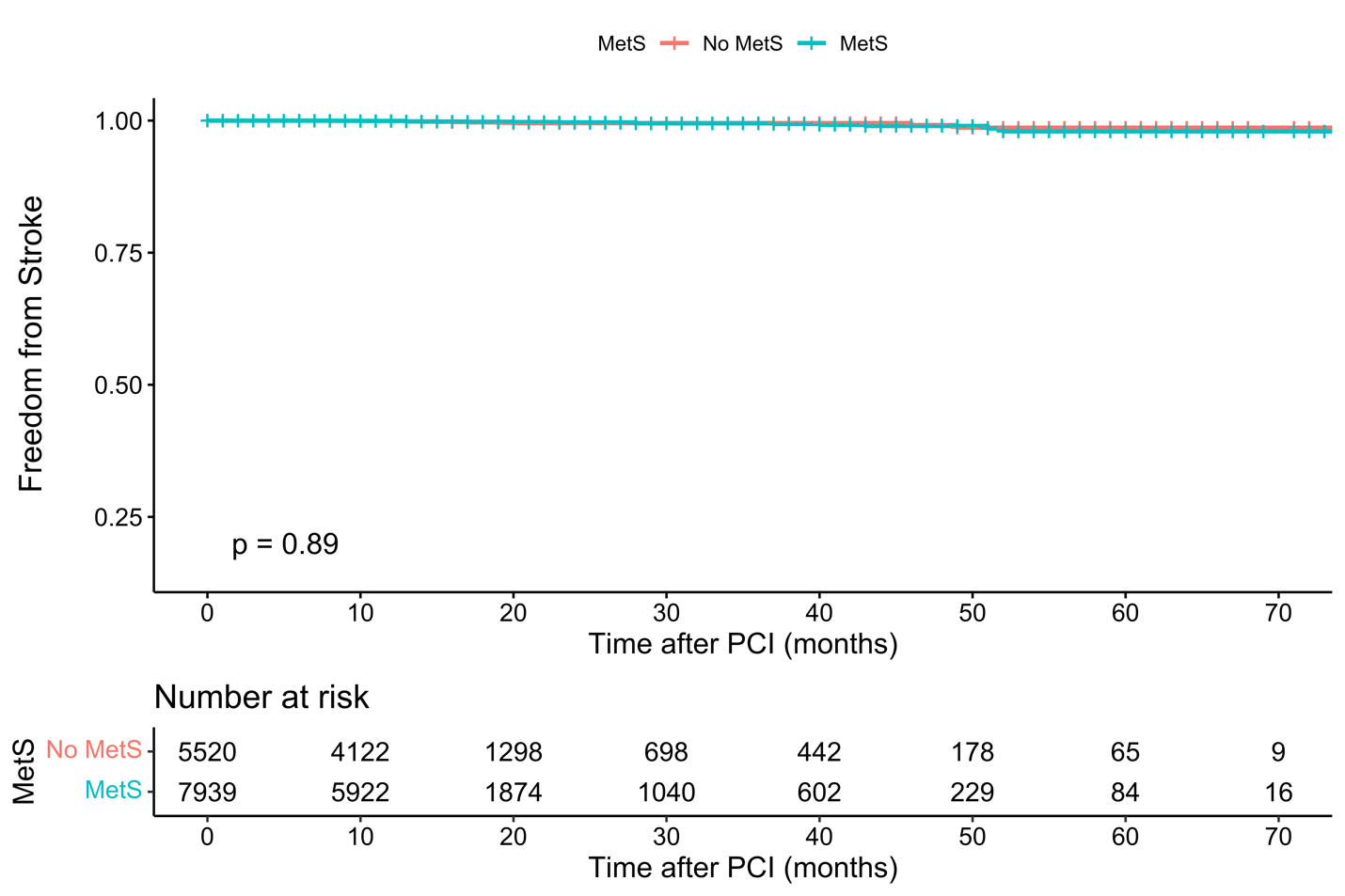


**Supplementary Figure 4.** Kaplan Meier for comparing stroke-free survival between MetS and non-MetS patients


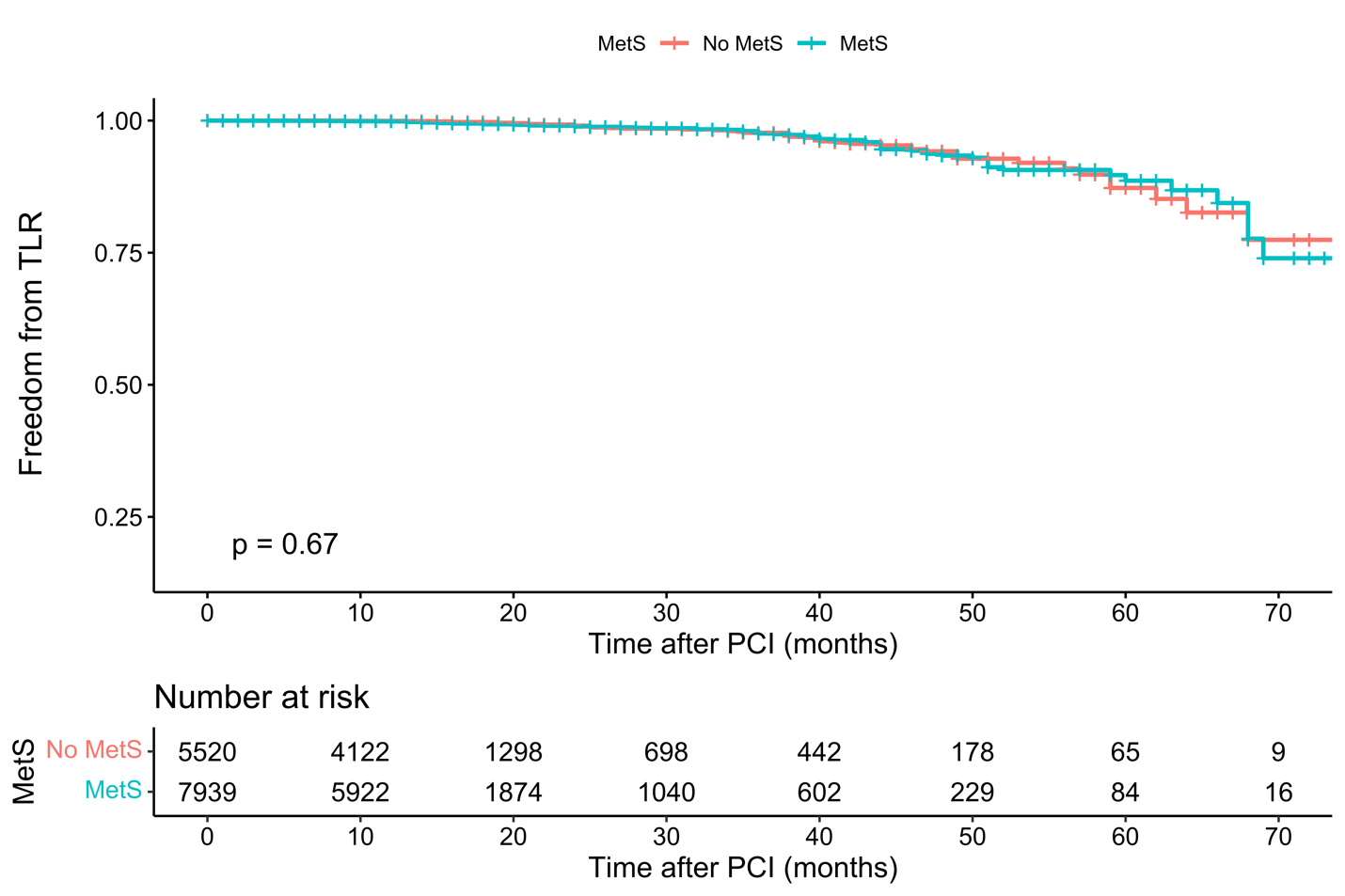


**Supplementary Figure 5.** Kaplan Meier for comparing target lesion revascularization-free survival between MetS and non-MetS patients


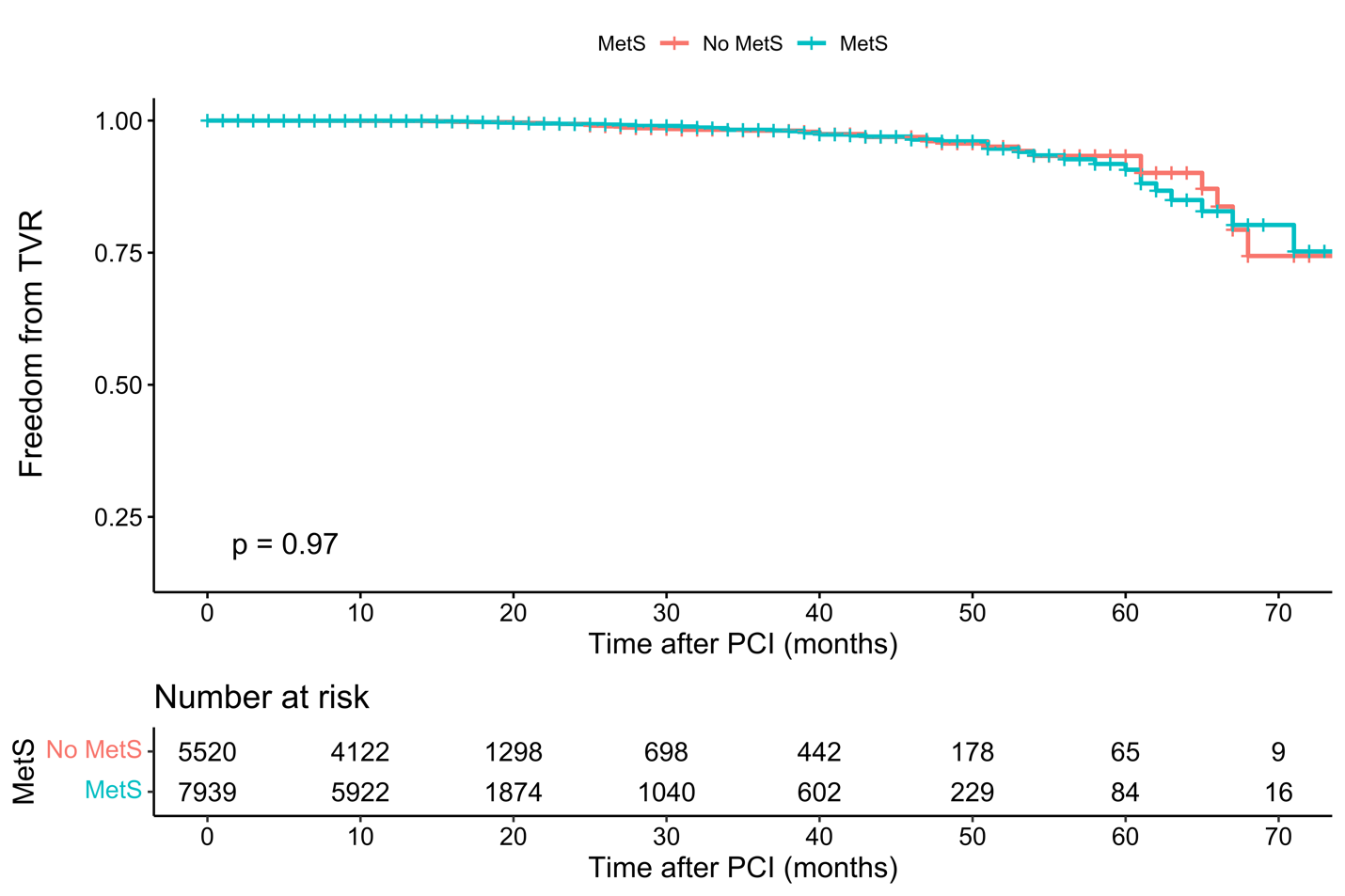


**Supplementary Figure 6.** Kaplan Meier for comparing target vessel revascularization-free survival between MetS and non-MetS patients
